# A new approach to analyse longitudinal epidemiological data with an excess of zeros

**DOI:** 10.1186/1471-2288-13-27

**Published:** 2013-02-20

**Authors:** Alette S Spriensma, Tibor RS Hajos, Michiel R de Boer, Martijn W Heymans, Jos WR Twisk

**Affiliations:** 1 Department of Epidemiology and Biostatistics, VU University Medical Center, P.O. Box 7057, Amsterdam, 1007 MB, The Netherlands; 2 EMGO Institute for Health and Care Research, VU University Medical Center, Van der Boechorststraat 7, Amsterdam, 1081 BT, The Netherlands; 3 Department of Methodology and Applied Biostatistics, Faculty of Earth and Life Sciences, Institute of Health Sciences, VU University, de Boelelaan 1085, Amsterdam, 1081 HV, The Netherlands; 4 Department of Medical Psychology, VU University Medical Centre, Van der Boechorststraat 7, Amsterdam, 1081 BT, The Netherlands; 5 Department of Health Sciences, University of Groningen, Antonius Deusinglaan 1, Groningen, 9713 AV, The Netherlands

**Keywords:** Two-part joint model, Excess of zeros, Count, Mixed modelling, Longitudinal, Statistical methods

## Abstract

**Background:**

Within longitudinal epidemiological research, ‘count’ outcome variables with an excess of zeros frequently occur. Although these outcomes are frequently analysed with a linear mixed model, or a Poisson mixed model, a two-part mixed model would be better in analysing outcome variables with an excess of zeros. Therefore, objective of this paper was to introduce the relatively ‘new’ method of two-part joint regression modelling in longitudinal data analysis for outcome variables with an excess of zeros, and to compare the performance of this method to current approaches.

**Methods:**

Within an observational longitudinal dataset, we compared three techniques; two ‘standard’ approaches (a linear mixed model, and a Poisson mixed model), and a two-part joint mixed model (a binomial/Poisson mixed distribution model), including random intercepts and random slopes. Model fit indicators, and differences between predicted and observed values were used for comparisons. The analyses were performed with STATA using the GLLAMM procedure.

**Results:**

Regarding the random intercept models, the two-part joint mixed model (binomial/Poisson) performed best. Adding random slopes for time to the models changed the sign of the regression coefficient for both the Poisson mixed model and the two-part joint mixed model (binomial/Poisson) and resulted into a much better fit.

**Conclusion:**

This paper showed that a two-part joint mixed model is a more appropriate method to analyse longitudinal data with an excess of zeros compared to a linear mixed model and a Poisson mixed model. However, in a model with random slopes for time a Poisson mixed model also performed remarkably well.

## Background

Within longitudinal epidemiological research, ‘count’ outcome variables frequently occur. Nowadays it is possible to analyse longitudinal ‘count’ outcome variables with advanced statistical techniques such as mixed models. Because ‘count’ data often follow a Poisson distribution, these data are mostly analysed with longitudinal Poisson regression. In many situations ‘count’ data does not exactly follow a Poisson distribution; they are often overdispersed, (i.e. the variance of the outcome variable is higher than the mean value). One of the solutions to deal with this overdispersion in count data is to use a negative binomial regression analysis [1]. However, overdispersion in the count variable is mostly caused by an excess of zeros, which cannot completely be controlled by assuming a negative binomial distribution. Examples of data with an excess of zeros (which are also known as ‘semicontinuous’ data) [2] within the field of epidemiology are: the number of hypoglycaemic events in diabetics, the number of hospitalisations in the general population, the number of sports injuries, the number of falls in a group of elderly people and the number of cigarettes smoked.

The classical methods to analyse outcome variables with an excess of zeros are to reduce the information in the data to either a dichotomous outcome variable (mostly comparing zero versus non-zero) or a categorical outcome variable (mostly comparing zero versus two groups of non-zero outcomes in which the groups are divided according to the median of the non-zero part). Sometimes, researchers try to transform (with a logarithmic transformation) a Poisson distribution with many zeros into a normally distributed variable. However, zeros cannot be log transformed and other computations such as adding ‘1’ to the ‘count’ outcomes with an excess of zeros before log transforming does not solve the problem either.

To properly address the problem of excess of zeros, several so-called two-part statistical models have been developed. These models, which are particularly popular in econometrics, are also known as mixed response or mixed distribution models and they include zero-inflated Poisson (ZIP) regression, zero-inflated negative binomial (ZINB) regression, sample selection methods, and hurdle models [3-16]. The idea behind these two-part approaches is that the outcome variable has a mixed distribution (i.e. a binomial distribution to deal with zero versus non-zero, and a Poisson (or other) distribution to deal with the non-zero part of the distribution). In the standard two-part approaches the two processes are split and for every process different regression coefficients are obtained. This also means that different sets of covariates can be included, one set for the binomial process (zero versus non-zero) and one set for the Poisson process. In a ZIP model, for instance, one regression coefficient reflects the relationship of a certain covariate with zero versus non-zero, while another regression coefficient reflects the relationship with the ‘count’ outcomes above zero. [17,18]. For some research questions (e.g. investigating the determinants of smoking behaviour) this is a nice feature. However, in many situations one regression coefficient for each covariate would be preferable (e.g. the analysis of hypoglycaemic events). Despite the preference of one regression coefficient, it should be realized that this regression coefficient is somewhat difficult to interpret, because it combines a binomial and a Poisson process into one coefficient. Models that provide one set of regression coefficients for the binomial distribution and Poisson (or other) distribution combined are known as two-part joint regression models [19-22]. For longitudinal data analysis these two-part joint regression models are almost never used in epidemiological practice.

The objective of this paper is to introduce a relatively ‘new’ method of a two-part joint mixed model (binomial/Poisson) in longitudinal data analysis for ‘count’ outcome variables with an excess of zeros. Furthermore, the performance of this new method will be compared to a linear mixed model and a Poisson mixed model; two models that are frequently used for longitudinal epidemiological data.

## Methods

### Dataset

The observational longitudinal dataset used for the analyses was obtained from the Study of the Psychological Impact in Real care of Initiating insulin glargine Treatment (SPIRIT) conducted between 2005 and 2009. This study aimed to examine the use of insulin glargine (a long acting insulin analog) on general emotional well-being, diabetes symptom distress and worries about hypoglycaemia in Dutch type 2 diabetes patients who previously used oral anti-hyperglycaemic medication. Type 2 diabetes patients who used oral anti-hyperglycaemic agents were recruited from 363 Dutch primary care practices, which were spread across the country. This resulted in a total sample of 889 patients. Measurements were conducted at baseline, after three and after six months. Results from this study have been presented previously [23]. We re-analysed the data in order to assess the development over time in hypoglycaemic events for diabetic patients, and the difference between low and high educated diabetes patients with three different mixed models.

### Statistical methods

All analyses were performed within the framework of longitudinal mixed models, The general idea behind mixed models for longitudinal data analysis is that an adjustment is made for the correlated outcome observations within individuals over time by estimating either the differences in average values of the outcome and/or the differences in relationships with time-dependent covariates. These differences i.e. variances are known as random effects and can be added to the intercept of the regression model (i.e. random intercept) and/or to the different regression coefficients of time-dependent covariates (i.e. random slopes) [24-26]. In this paper two ‘standard’ approaches, i.e. a linear mixed model treating the outcome variables as normally distributed and a Poisson mixed model treating the outcome variables as Poisson distributed, will be compared with a two-part joint regression model in order to analyse the development over time and to analyse the differences between low and high educated patients. Equation 1a shows the linear mixed model with only a random intercept, while equation 1b shows the linear mixed model with both a random intercept and a random slope for time.

(1a) $$y_{\mathrm{ij}}=\left(\beta_{1}+\zeta_{1j}\right)+\beta_{2}x_{\mathrm{ij}}+\in_{\mathrm{ij}}$$

(1b) $$y_{\mathrm{ij}}=\left(\beta_{1}+\zeta_{1j}\right)+\left(\beta_{2}+\zeta_{2j}\right)x_{\mathrm{ij}}+\in_{\mathrm{ij}}$$

Where *y*_*ij*_ is the hypoglycaemic score for the j^th^ patient at the i^th^ time, *x*_*ij*_ is the corresponding time, *β*_1_ the fixed intercept of the patients,*ζ*_1*j*_ the patient-specific random intercept, *β*_2_ the fixed slope of the patients, *ζ*_2*j*_ the patient-specific random slope, and ζ_*ij*_ is a patient-specific residual error term at the i^th^ time [26]. It was assumed that each of the two variations in the random intercept and the random slope was normally distributed with an average of zero and a variance *σ*^2^. Furthermore, the Poisson (ln(*μ*_*ij*_)) mixed model can be specified in a similar way.

For the two-part joint approach, a binomial/Poisson mixed distribution was used. The general idea behind this mixture is that the outcome variable has a binomial distribution for the zero versus non-zero part and a Poisson distribution for the non-zero part. The binomial distribution is modelled by a logit link function, while the Poisson distribution is modelled by a log link function. The response probability of a longitudinal two-part joint binomial/Poisson regression model can be written down as:

(2) $$\mathrm{Pr}\left(y_{\mathrm{ij}}|x_{\mathrm{ij}}\right)=\pi_{1}g\left(y_{\mathrm{ij}};\mu_{\mathrm{ij}}=0\right)+\pi_{2}g\left(y_{\mathrm{ij}};\mu_{\mathrm{ij}}=\exp\left({x^{\prime }}_{\mathrm{ij}}\beta \right)\right)$$

The first part of the equation has a mean of zero and the second part of the equation has a mean that depends on the covariates (time). *π*_1_ and *π*_2_=1−*π*_1_ are the component weights/latent class probabilities and g(*y*_*ij*_;*μ*_*ij*_) is the Poisson probability for count *y*_*ij*_ with mean *μ*_*ij*_[27]. A full explanation of the mathematical background of the analyses with mixed distribution models can be found in other papers [28-36]. For the two-part joint model, random intercepts and random slopes can be added in a similar fashion as for the linear mixed model.

In the present analyses, educational level was modelled as a dichotomous variable distinguishing between low and high education (with low education as reference), time was modelled as a categorical variable (represented by two dummy variables, with baseline as reference). Two model fit parameters were used to compare the three models with each other. Firstly, the Bayesian information criterion (BIC) was used. The BIC is an indicator of model fit, based on the −2 log likelihood, but taking into account the number of parameters estimated [37]. A lower BIC indicates a better performance of the model. Secondly, predicted frequencies (including the random effects) of the outcome variable, obtained when fitting the models, were compared to observed frequencies in hypoglycaemic events to compare the accuracy of the different models. This comparison was graphically presented in scatter plots. In addition, the means of the squared residuals (MSR) were computed for the different models. A lower MSR indicates a better performance of the model.

All analyses were performed with Stata (version 11.1) [38]. Estimations were performed with the GLLAMM procedure [26,39], using adaptive quadrature to estimate the random effects. Scatter plots were created within PASW Statistics 18 [40].

## Results

**Figure 1:**
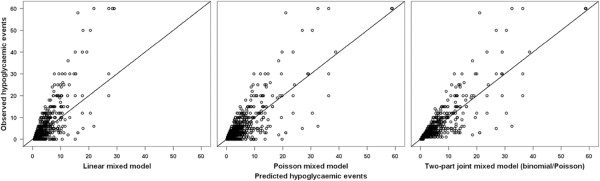
Scatter plots of the observed vs. predicted values for the three longitudinal models with a random intercept, evaluating the hypoglycaemic events for education.

Table 1 shows the number, the proportion, and the median of the patients who have experienced ≥1 hypoglycaemic event for the three measurements over time by education as well as for the total number of patients. The proportion of both lower and higher educated patients that experienced ≥1 hypoglycaemic event increased over time. 34.5% of the lower educated patients experienced ≥1 hypoglycaemic event at baseline and after six months this increased to 37.9%, for the higher educated patients the percentage increased from 43.1% to 50.4%. In contrast, the median number of events for subjects with ≥1 hypoglycaemic event decreased over time. For the lower educated patients the median decreased from 4 to 2 and for the higher educated patients from 4 to 3. Table 2 shows the results of the analyses relating the hypoglycaemic events (dependent variable) to educational level (independent variable) when the number of hypoglycaemic episodes was treated as normal, Poisson or binomial/Poisson (two-part joint) distributed. All three models showed a significant positive relationship between education and the number of hypoglycaemic events. The model fit was best for the two-part joint mixed model (binomial/Poisson) (BIC: 6687.64, MSR: 7.26). Furthermore, Figure 1 depicts the accuracy of the different analyses in scatter plots of observed vs. predicted values. The binomial/Poisson model clearly performed best especially in correctly predicting the number of patients with zero events.

**Table 1:** The proportion and median of diabetes patients with ≥ 1 hypoglycaemic event by time and educational level*

	**Low education**			**High education**			**Total**		
**≥ 1 hypoglycaemic events**	**n**	**Percentage**	**Median (≥ 1 hyp)**	**n**	**Percentage**	**Median (≥ 1 hyp)**	**n**	**Percentage**	**Median (≥ 1 hyp)**
T0 (Baseline)	140	(34.5%)	4	157	(43.1%)	4	297	(38.6%)	4
T1 (3 months)	121	(36.9%)	3	141	(49.3%)	3	262	(42.7%)	3
T2 (6 months)	105	(37.9%)	2	117	(50.4%)	3	222	(43.6%)	3

* Having zero hypoglycaemic events is the complement of ≥ hypoglycaemic events.

**Table 2:** Regression and model fit parameters for the three longitudinal models with a random intercept, evaluating the difference in hypoglycaemic events for education*

	**Linear mixed model**			**Poisson mixed model**			**Two-part joint mixed model (binomial/Poisson)**		
	**Coef.**	**Std. Err.**	**P > |z|**	**Coef.**	**Std. Err.**	**P > |z|**	**Coef.**	**Std. Err.**	**P > |z|**
Educational level (high education)	1.14	(0.36)	0.001	0.66	(0.15)	0.000	0.62	(0.15)	0.000
BIC	11842.87			7609.073			6687.639		
Mean squared residual	13.75			9.73			7.26		

Abbreviation: Regression coefficients (Coef.), Standard errors (Std. Err.), P-value (P > |z|), Bayesian information criterion (BIC).

* Education is time independent, therefore random slopes could not be calculated.

**Figure 2:**
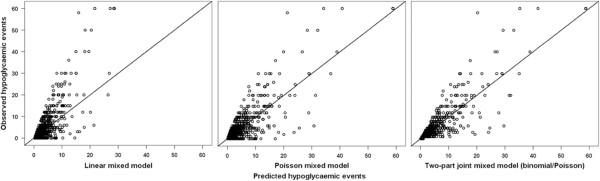
Scatter plots of the observed vs. predicted values for the three longitudinal models with only a random intercept, evaluating the difference in development of the hypoglycaemic events over time.

**Figure 3:**
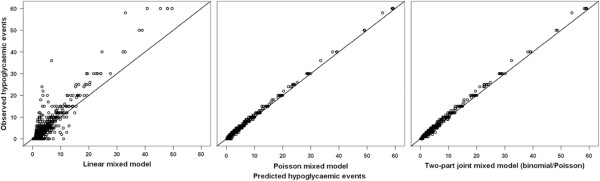
Scatter plots of the observed vs. predicted values for the three longitudinal models with a random intercept and random slopes for time, evaluating the difference in development of the hypoglycaemic events over time.

Table 3 shows the results of the analyses regarding the development over time as independent variable with only a random intercept. In all three models the regression coefficients for time were negative, and the corresponding P-values at T2 were significant. Comparing both the fit indicators (Table 3) and the accuracy (Figure 2), similar results were found as for the analyses comparing higher and lower educated patients. The two-part joint model (binomial/Poisson) had the best model fit (BIC: 7013.64, MSR: 6.56) and was also best in correctly predicting the zero events. However, the models changed considerably once random slopes for time were added to the models (Table 4): The signs of the regression coefficients for the Poisson mixed model and the two-part joint mixed model (binomial/Poisson) changed from negative to positive. The regression coefficients derived from the Poisson mixed model changed from −0.11 (3 months) and −0.26 (6 months) to 0.28 (3 months) and 0.38 (6 months) when random slopes were included. For the two-part joint mixed model (binomial/Poisson) the regression coefficients changed from −0.18(3 months) and −0.27 (6 months) to 0.12 (3 months) and 0.25 (6 months). Adding random slopes to the models resulted in a much better fit for the Poisson (BIC: 6774.75, MSR: 0.24) and the two-part joint mixed model (binomial/Poisson) (BIC: 6467.55, MSR: 0.30). Furthermore, the predicted values (Figure 3) for the Poisson mixed model and the two-part joint mixed model (binomial/Poisson) were in close accordance to the observed values. However, to a small extent there was still a difference in the correctly estimated zeros in favour of the two-part joint mixed model (binomial/Poisson). In total, 89.5% of the zeros were correctly estimated for the Poisson mixed model and 92.8% of the zeros were correctly estimated for the longitudinal two-part joint mixed model.

**Table 3:** Regression and model fit parameters for the three longitudinal models with a random intercept, evaluating the difference in development of the hypoglycaemic events over time

	**Linear mixed model**			**Poisson mixed model**			**Two-part joint mixed model (binomial/Poisson)**		
	**Coef.**	**Std. Err.**	**P > |z|**	**Coef.**	**Std. Err.**	**P > |z|**	**Coef.**	**Std. Err.**	**P > |z|**
T1 (3 months)	−0.31	(0.26)	0.220	−0.11	(0.11)	0.333	−0.18	(0.12)	0.129
T2 (6 months)	−0.73	(0.24)	0.002	−0.26	(0.11)	0.015	−0.27	(0.10)	0.010
BIC	12395.09			7983.639			7013.644		
Mean squared residual	13.61			9.02			6.56		

Abbreviation: Regression coefficients (Coef.), Standard errors (Std. Err.), P-value (P > |z|), Bayesian information criterion (BIC).

**Table 4:** Regression and model fit parameters for the three longitudinal models with a random intercept and random slopes for time, evaluating the difference in development of the hypoglycaemic events over time

	**Linear mixed model**			**Poisson mixed model**			**Two-part joint mixed model (binomial/Poisson)**		
	**Coef.**	**Std. Err.**	**P > |z|**	**Coef.**	**Std. Err.**	**P > |z|**	**Coef.**	**Std. Err.**	**P > |z|**
T1 (3 months)	−0.35	(0.27)	0.201	0.28	(0.16)	0.093	0.12	(0.15)	0.424
T2 (6 months)	−0.82	(0.26)	0.001	0.38	(0.17)	0.027	0.25	(0.16)	0.128
BIC	12198.49			6774.745			6467.549		
Mean squared residual	5.12			0.24			0.30		

Abbreviation: Regression coefficients (Coef.), Standard errors (Std. Err.), P-value (P > |z|), Bayesian information criterion (BIC).

## Discussion

This study showed that the two-part joint mixed model (binomial/Poisson) model performed much better than the ‘conventional’ mixed models when only a random intercept was added to the models. This was especially the case in estimating the excess of zeros. However, when random slopes were added to the models, performance of the Poisson mixed model increased considerably and performed more or less the same as the two-part joint mixed model (binomial/Poisson).

It is known from the literature that Poisson regression can handle even a high fraction of zeros [1]. In the present study the percentage of subjects having zero events was relatively high and decreased over time from 61.4% to 56.4%. However, it is not exactly known to what extent the Poisson distribution would be able to model the excess of zeros. In addition, the performance of the Poisson mixed model regarding the number of zeros improved considerably when random slopes were added to the model. Surprisingly, adding random slopes to the model resulted not only in a much better fit, but also in a sign change for the development over time in both the Poisson mixed model and the two-part joint mixed model (binomial/Poisson). Although is it not clear why this sign change occurs, a possible explanation can be that in a model with a random intercept, only the ‘average’ values are allowed to differ between the subjects and therefore the regression coefficient obtained from these analyses only reflects the ‘average’ decrease in the number of events. When adding random slopes to the models, also the development over time is allowed to differ between the subjects. Therefore, an analysis with both a random intercept and random slopes also reflects the increased probability of having an event. This leads to a much better fit and a positive regression coefficient instead of an inverse one.

The interpretation of the regression coefficients of the linear mixed model and the Poisson mixed model are quite straightforward. For example, the interpretation of the relation between education and hypoglycaemic events can be interpreted as following for the linear mixed model (Table 2): Higher educated diabetic patients have 1.14 more events than (on average over time) compared to lower educated diabetic patients. For the Poisson mixed model the regression coefficient can be interpreted as (Table 2): exp (0.66) = 1.93. On average over time, higher educated diabetic patients have an increased prevalence rate of 93% in hypoglycaemic events compared to lower educated diabetic patients. The interpretation of the regression coefficient of the two-part joint mixed model is somewhat more complicated, since the model gives a combined regression coefficient for the binomial process and the Poisson process. However, some researchers have interpreted the regression coefficient of a two-part joint model as being the result for the cases that are above the limit [41] p. 320, [42] p. 503. These cases above the limit would be interpreted in the same way as a Poisson model i.e. higher educated diabetic patients have an increased prevalence rate of 86% in hypoglycaemic events compared to lower educated diabetic patients (exp(0.62) = 1.86). To overcome the problem of the interpretation of a combined regression coefficient, McDonald and Moffit [41] have developed a decomposition technique for the regression coefficient of a two-part joint binomial/normal (tobit) model. The general idea of their decomposition technique is that the regression coefficient combines two interpretations: 1) The difference in the outcome variable of being above the limit, weighted by the probability of being above the limit; and 2) the difference in the probability of being above the limit, weighted by the expected value of the outcome variable if above the limit [43]. In theory, this technique could also be used for two-part joint models that, instead of using a normal distribution, use another distribution such as the Poisson distribution for the values that are above zero.

In the present paper a two-part joint model was used to model the number of hypoglycaemic events obtaining a shared regression coefficient for both the binomial and the Poisson distribution combined. An important reason why one regression coefficient is preferred is that the outcome variable in this example (i.e. the number of hypoglycaemic events) should be seen as one process that cannot be split into two processes with separate regression coefficients. In contrast, sometimes it is better to analyse the data with a two-part separate model, leading to separate regression coefficients for both parts of the process. An example could be the analysis of determinants of smoking behaviour, which can be different for the logistic part of the analysis and the Poisson part. The logistic part of the analysis may need a set of covariates in order to model why some people smoke and others do not smoke, furthermore a different set of covariates may be needed to model how many cigarettes a person will smoke.

## Conclusions

This paper showed that the two-part joint mixed model (binomial/Poisson) is a more appropriate method for the analysis of longitudinal data with an excess of zeros when only a random intercept is included into a model. However, in the model with random slopes for time, also the Poisson mixed model performed remarkably well. In addition, more research is needed on the interpretation of the regression coefficients of the longitudinal two-part joint model.

## Competing interests

The authors declare that they have no competing interests.

## Authors’ contributions

ASS made contributions to the design, conducted the analysis and interpretation of the data, and drafted the manuscript. TRSH made contributions to the acquisition of the data, and reviewed the article critically for important intellectual content. MRB made contributions to the interpretation of the data, and reviewed the article critically for important intellectual content. MWH made contributions to the interpretation of the data, and reviewed the article critically for important intellectual content. JWRT made substantial contributions to the conception and design and the analysis of data, helped to draft the manuscript, supervised the analysis and interpretation of the data, and reviewed the article critically for important intellectual content. All authors read and approved the final manuscript.

## Pre-publication history

The pre-publication history for this paper can be accessed here:

http://www.biomedcentral.com/1471-2288/13/27/prepub
